# Monitoring of molecular responses to tirabrutinib in a cohort of exceptional responders with relapsed/refractory mantle cell lymphoma

**DOI:** 10.1002/jha2.966

**Published:** 2024-06-24

**Authors:** Abdullah N. M. Alqahtani, Sandrine Jayne, Matthew J. Ahearne, Christopher S. Trethewey, Sai S. Duraisingham, Susann Lehmann, Caroline M. Cowley, Martin J. S. Dyer, Harriet S. Walter

**Affiliations:** ^1^ The Ernest and Helen Scott Haematological Research Institute, Leicester Cancer Research Centre, Department of Genetics and Genome Biology, University of Leicester Leicester UK; ^2^ Department of Haematology University Hospitals Leicester Leicester UK; ^3^ Cancer Molecular Diagnostic Laboratory (CMDL) University of Cambridge Cambridge UK; ^4^ Department of Immunology University Hospitals Leicester Leicester UK; ^5^ Institute for Precision Health, Leicester Drug Discovery and Diagnostics, University of Leicester Leicester UK; ^6^ Department of Genetics and Genome Biology Leicester Molecular Diagnostics Leicester Cancer Research Centre, University of Leicester Leicester UK; ^7^ Department of Oncology University Hospitals Leicester Leicester UK

To the Editor,

Inhibitors of Bruton's tyrosine kinase (BTK) in chronic lymphocytic leukemia (CLL) result in durable responses in nearly all patients [1]. In contrast, in more aggressive B‐cell malignancies, including diffuse large B cell lymphoma (DLBCL) and mantle cell lymphoma (MCL), responses to single agent BTK inhibitors (BTKi) occur only in subsets of patients and are mostly of brief duration. However, exceptional responses may occur [2, 3, 4, 5, 6].

In relapsed/refractory (R/R) MCL, median progression‐free survival (PFS) with single‐agent covalent BTKi range from 12 (ibrutinib) to 20 months (acalabrutinib ACE‐LY‐004 NCT02213926 study) (Table S1) [7, 8, 9]. Analysis of responding patients in ACE‐LY‐004 showed that 8/29 evaluable patients eradicated minimal residual disease (MRD) using the quantitative ClonoSEQ next‐generation sequencing assay of cellular peripheral blood DNA [10]. However, molecular determinants of responsiveness to BTKi in MCL, and clinical significance of attaining MRD negativity in this setting remain unknown.

Like acalabrutinib, tirabrutinib is a highly selective BTKi, binding covalently to BTKC481 via a reactive acyl alkyne group. Although only 16 MCL patients were treated with single‐agent tirabrutinib within the POE001 phase 1 trial (NCT01659255), extended follow‐up showed an estimated median PFS of 25.8 months at 3 years [11]. Three patients from this cohort, described below, attained complete responses lasting over 72 months, despite adverse prognostic features at diagnosis including *TP53* mutations, blastoid morphology, and refractoriness to immunochemotherapy. In these exceptional responders [12], we sought to determine common features or a tumoral mutational signature that might predict exceptional responsiveness. Secondly, we determined the depth of response using digital droplet PCR (ddPCR) in both plasma and cellular DNA samples and whether serial assessment of levels of cellular and plasma circulating tumor (ct) DNA samples might presage relapse.

Clinical and laboratory details of the three cases are given in Table 1. A schema of the treatment timelines is shown in Figure 1. Full materials and methods are found in the Supporting Information. The three patients (201‐139, 201‐162, and 201‐170) were all treated at our center and received either 480 or 600 mg of tirabrutinib once per day. All entered a clinical and radiological remission. One patient (patient 201‐162) with primary immunochemotherapy‐refractory disease remains in complete remission (CR) > 108 months from trial initiation. However, in 2020, they developed estrogen receptor positive grade 2 invasive ductal breast cancer, necessitating temporary discontinuation of tirabrutinib for 4 months. The breast cancer was treated radically with surgical resection, post operative radiotherapy and tamoxifen. There remains no clinical or radiological evidence of recurrence. The other two patients relapsed 91 and 72 months after initiating tirabrutinib. Due to comorbidities, no further therapy was attempted in patient 201‐170 and the patient died rapidly from uncontrolled disease. Patient 201‐139 received rituximab, bendamustine, cytosine arabinoside, and subsequently pirtobrutinib with the aim of proceeding to chimeric antigen receptor (CAR)‐T cell therapy, but failed to respond adequately.

**TABLE 1:** Clinical data from the three exceptional responder mantle cell lymphoma (MCL) patients.

Patient ID	201‐139	201‐162	201‐170
**M/F**	M	F	F
**Age at diagnosis**	60	64	69
**MIPI score**	High	Intermediate	High
**Age at initiation of Tirabrutinib**	63	65	73
**Dose of Tirabrutinib**	480 mg	600 mg	600 mg
**Number of prior lines**	3	2	2
**Relapsed/Refractory**	Refractory	Refractory	Refractory
**Prior allogeneic or autologous SCT?**	Y—allogeneic	N	N
**LN > 5.0 cm?**	Y	Y	Y
**Lymphocytosis?**	N	N	N
**Ki67**	50%	20%–30%	80%
**Duration of response (months)**	72	Ongoing CR at 108 months	91
**Status**	Deceased	Alive and continues Tirabrutinib	Deceased

**FIGURE 1:**
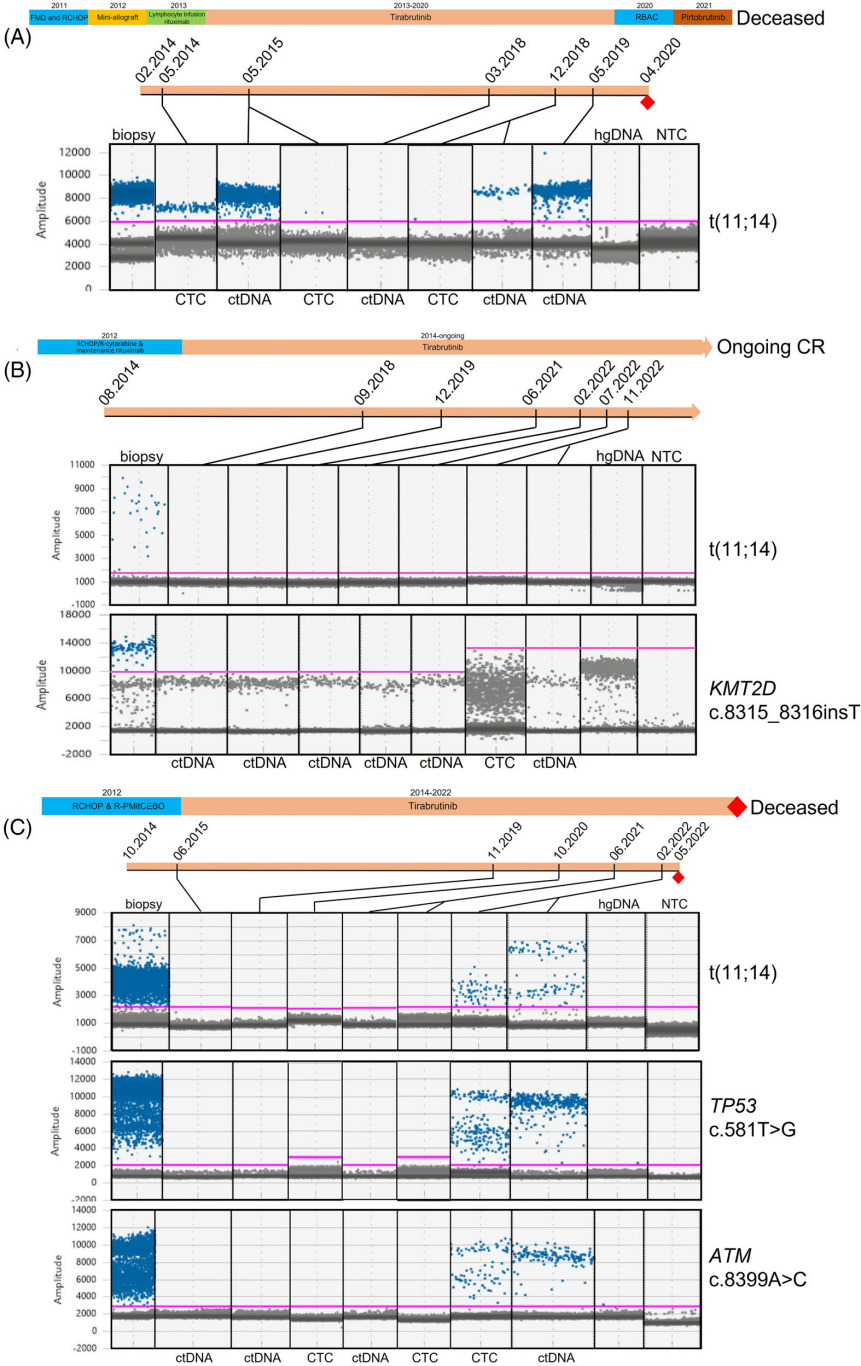
(A) Treatment timelines for the three exceptional responder patients. Red diamonds correspond to the time of clinical progression, (B–D) Droplet frequency plots corresponding to serial ddPCR analysis during treatment with tirabrutinib (timeline) performed for patient 201‐139 detected with t(11;14) translocation breakpoint assay (B), for patient 201‐162 with t(11;14) translocation breakpoint (top panel) and *KMT2D* (c.8315_8316insT) (bottom panel) assays (C), for patient 201‐170 with t(11;14) translocation breakpoint (top panel), *TP53* (c.581T > G) (middle panel) and *ATM* (c.8399A > C) (bottom panel) assays (D) with additional biopsy positive and human genomic DNA (hgDNA) negative controls. The pink line indicates amplitude thresholds. Mutant droplets are indicated in blue; negative droplets in grey. ctDNA, circulating tumor DNA; CTC, circulating tumor cells; NTC, no template control. ddPCR assays were run at different time points and figures were constructed for clarity.

Whole exome sequencing showed t(11;14)(q13;q32) with breakpoints in *CCND1* and *IGH* as anticipated, but there were no other common mutations (Table S2). Two patients exhibited *TP53* mutation (pL194R and pR181H), one *KMT2D* mutation (p.S2773Lfs*72), and one patient had two *ATM* mutations. All three cases showed unmutated (> 99% homology to germline) *IGHV* gene segment usage; two cases utilized *IGHV4‐34*, the other *IGHV3‐23*. Unmutated *IGHV4‐34* is seen in 15% of MCL and in 15% of the non‐germinal center subtype of DLBCL, including the MCD subgroup, sensitive to BTKi. Unmutated IGHV4‐34 recognizes autoantigens on the surface of malignant B cells, shown to result in chronic active BCR signaling in activated B‐cell‐like DLBCL models [13]. At relapse, in patient 201‐139 mutations in *PLCG2* (p.M1141T, 54% VAF), previously described in relapsed CLL with ibrutinib, and in *NFKB2* (p.G373_G374insEGVLC, 36% VAF) that activates the alternative nuclear factor κappa B pathway were seen. No *BTK* mutations were present, supporting prior findings [14].

Interestingly, in all 3 cases, we observed a complete absence of peripheral blood CD19+ B cells with tirabrutinib, consistent with B cell aplasia. In one case (201‐139) bone marrow analysis confirmed a B‐cell differentiation block at the CD19+ CD38+ sIg‐ B‐cell precursor stage (Figure S1A). In this patient, B cell aplasia resulted in hypogammaglobulinemia and recurrent encapsulated bacterial infections commencing 30 months following the initiation of tirabrutinib (Figure S1B). No hypogammaglobulinemia nor infections were observed in the other two patients. This phenotype was not observed in this cohort in those with a shorter duration or depth of response.

From sequencing data, we derived patient‐specific ddPCR assays to monitor both t(11;14)(q13;q32) translocation breakpoint and point mutations (Table S3). In serial cellular and plasma DNA samples, all cases initially showed clearance of tumor DNA from the peripheral blood either as ctDNA or genomic (g) DNA derived from circulating tumor cells (CTC) (Figure 1A–C and Figure S2). In the two relapsing patients it was possible to detect recurrent tumor DNA in peripheral blood either as plasma ctDNA or CTC genomic DNA (gDNA), 3 months, and 14 months, prior to radiological and clinical relapse. In patient 201‐162, the disease remains undetectable. *BTK* C481S, T474I, or L528W mutations were not detectable using ddPCR in all three patients (data not shown).

Although our data are limited by a small sample size, the three exceptional responder cases who entered durable CR with single agent BTKi lacked a common mutational signature and genetic evidence for chronic active BCR signaling. These findings identify with Wheeler et al., 2021 where the molecular basis for therapeutic success in exceptional responders was identified in less than one quarter of 111 exceptional responders [12].

We show that serial monitoring of plasma ctDNA and CTC gDNA samples by ddPCR demonstrated early detection of progressive disease in the context of a BTKi treatment. Similar to prior studies, tumor‐specific clonotypes can be identified in MCL enabling tracking in peripheral blood. In both relapsing patients, ctDNA was more sensitive than the cellular DNA‐based assay in the detection of disease re‐emergence ahead of radiological and clinical relapse. These data might have permitted early initiation of CAR‐T therapy before the development of rapidly progressive, overwhelming disease; some 40% of patients with R/R MCL may fail to get to CAR‐T therapy due to such rapid progression [15].

## AUTHOR CONTRIBUTIONS

Abdullah N. M. Alqahtani, Sandrine Jayne, Christopher S. Trethewey, Sai S. Duraisingham, and Susann Lehmann performed research. All authors were involved in data analysis and interpretation. Harriet S. Walter, Sandrine Jayne, and Martin J. S. Dyer drafted the article that was revised and approved by all authors (article writing). Martin J. S. Dyer and Harriet S. Walter designed and supervised the study.

## CONFLICT OF INTEREST STATEMENT

Martin J. S. Dyer has received research funding from Gilead Sciences. Harriet S. Walter has received research funding from Gilead Sciences. Sandrine Jayne has received research funding from Gilead Sciences. All the other authors declare no conflict of interest.

## FUNDING INFORMATION

This work was supported by funds from King Faisal Medical City for Southern Regions (KFMCity) (Saudi Arabia), the Scott‐Waudby Trust, the Hope Against Cancer charity, Cancer Research UK in conjunction with the UK Department of Health on an Experimental Cancer Medicine Centre grant [C10604/A25151] and by Leicester Drug Discovery and Diagnostics under the University of Leicester Institute for Precision Health [MRC—Impact Acceleration Account MR/X502777/1]. This research used the Core Biotechnology Services (NUCLEUS) and the ALICE High Performance Computing Facility at the University of Leicester. The research was carried out at the National Institute for Health and Care Research (NIHR) Leicester Biomedical Research Centre (BRC).

## ETHICS STATEMENT

This study was approved by the local Research Ethics Committee and the University Hospitals of Leicester Nation Health Service Trust (06/Q2501/122). All patients were consented to this.

## PATIENT CONSENT STATEMENT

The authors have confirmed patient consent statement is not needed for this submission.

## CLINICAL TRIAL REGISTRATION

The authors have confirmed clinical trial registration is not needed for this submission.

## Supporting information


**FIGURE S1** Development of B cell aplasia in patients treated with tirabrutinib. (A) Multicolor flow cytometry of bone marrow from healthy control (top), patient 201‐139 during treatment (middle) and at relapse (bottom) for expression of CD45, CD20, CD19, CD38 showing the B‐cell differentiation block at the CD19+ CD38+ sIg‐ B‐cell precursor stage and the absence of cells expressing kappa or lambda light chains (B) Development of hypogammaglobulinemia, immunoglobulins concentration (left panel) and lymphoid cells counts (right panel) in patient 201‐139 during treatment.

**FIGURE S2** Serial wild‐type ddPCR analysis was performed on *CCND1* wildtype at the site of t(11;14) translocation breakpoint for patient 201‐139 (A), for patient 201‐162 (top panel) (B) and for patient 201‐170 (top panel) (C). Additionally, ddPCR analysis was performed for patient 201‐162 on *KMT2D* (bottom panel) (B), and for patient 201‐170 on *TP53* (middle panel) and *ATM* (bottom panel) (C) corresponding wild‐type sequences with additional biopsy and human genomic DNA positive controls. Wildtype droplets are indicated in green; negative droplets are grey. NTC, no template control; ctDNA; circulating tumor DNA; CTC: circulating tumor cells. ddPCR assays were run at different time points and figures were constructed for clarity.

Supporting Information

Supporting Information

## Data Availability

Data are available on request from the authors. The data that support the findings of this study are available from the corresponding author upon reasonable request.
