# Supplementary figures and images for: Monitoring of molecular responses to tirabrutinib in a cohort of exceptional responders with relapsed/refractory mantle cell lymphoma
Source: EJHaem. 2024 Jun 24;5(4):896–9. doi: 10.1002/jha2.966 (PMC11327755; doi:10.1002/jha2.966)

# A

HEALTHY  
CONTROL

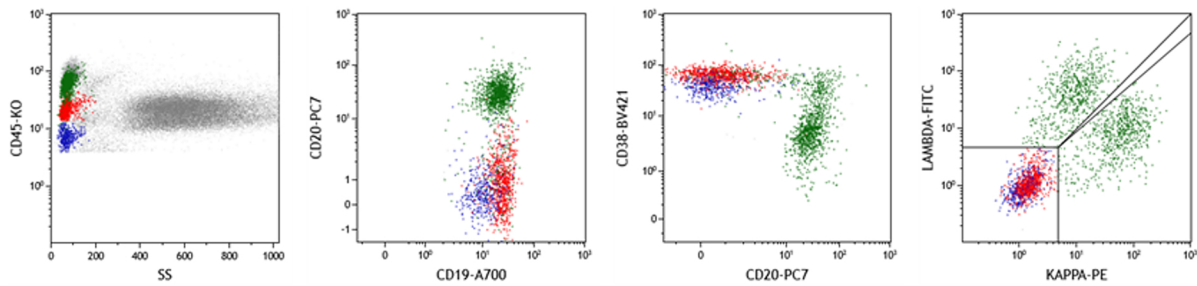

201-139  
During  
Tirabrutinib

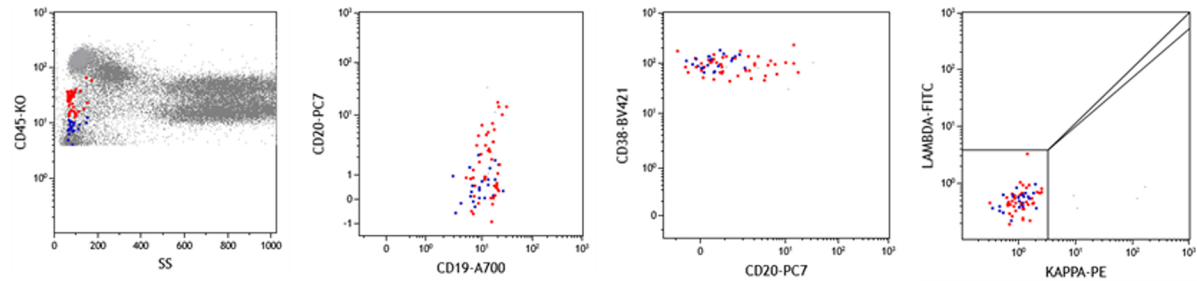

201-139  
Relapse

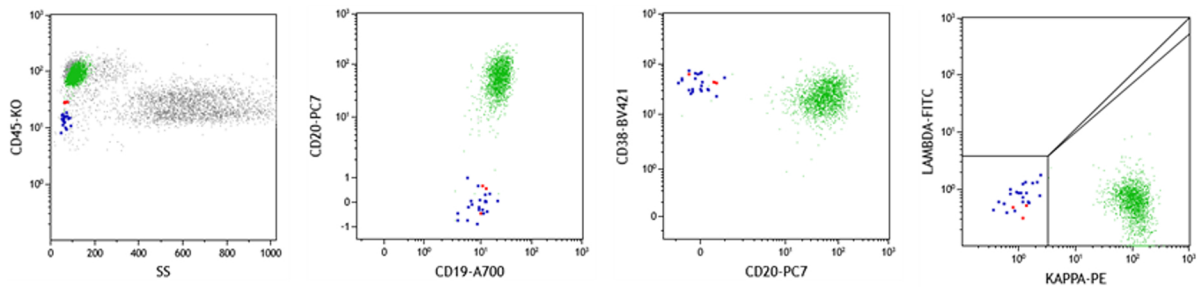

# B

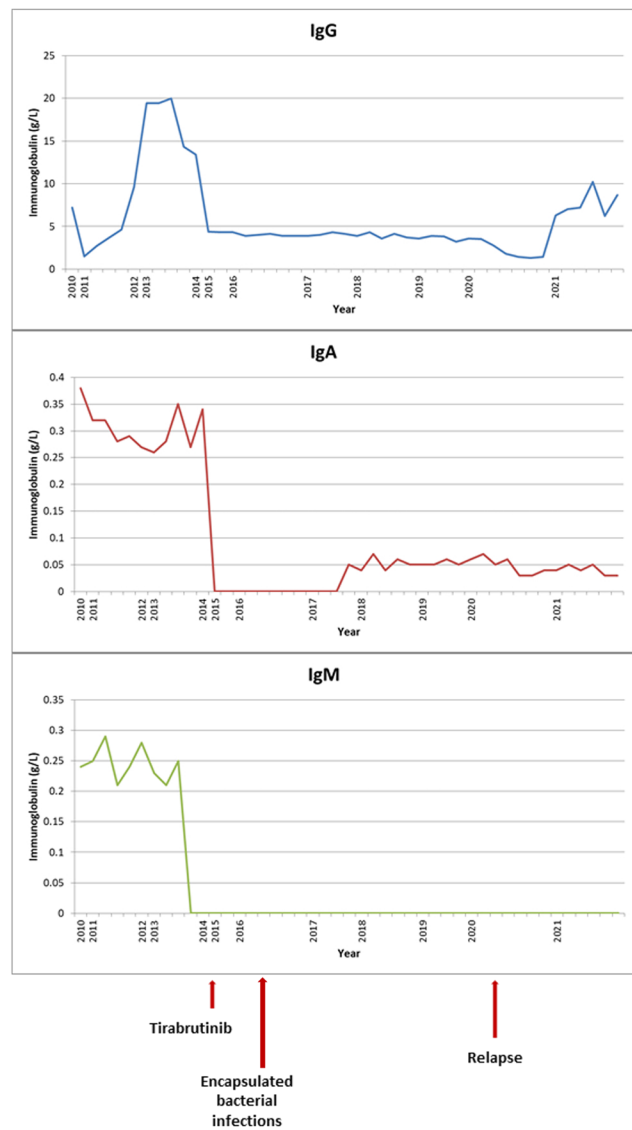

# C

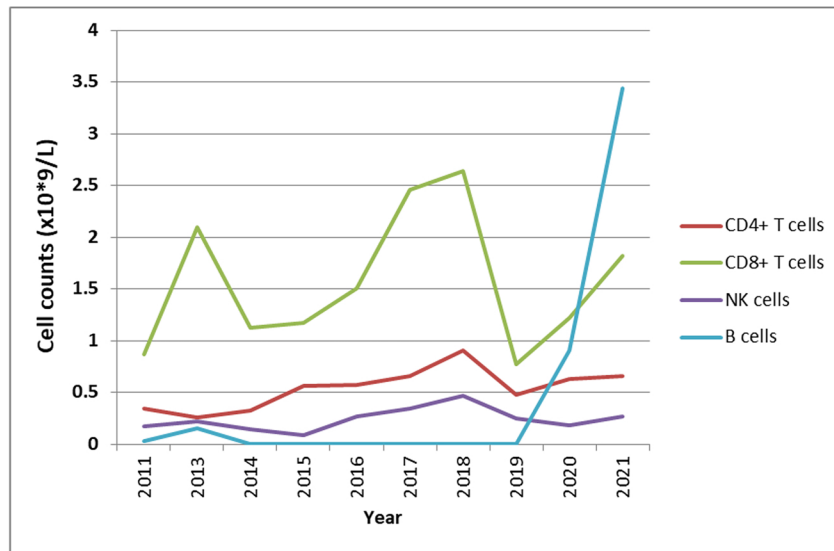

Supplement: Supplementary file 1 — FIGURE S1 Development of B cell aplasia in patients treated with tirabrutinib. (A) Multicolor flow cytometry of bone marrow from healthy control (top), patient 201‐139 during treatment (middle) and at relapse (bottom) for expression of CD45, CD20, CD19, CD38 showing the B‐cell differentiation block at the CD19+ CD38+ sIg‐ B‐cell precursor stage and the absence of cells expressing kappa or lambda light chains (B) Development of hypogammaglobulinemia, immunoglobulins concentration (left panel) and lymphoid cells counts (right panel) in patient 201‐139 during treatment. [file JHA2-5-896-s002.pdf]

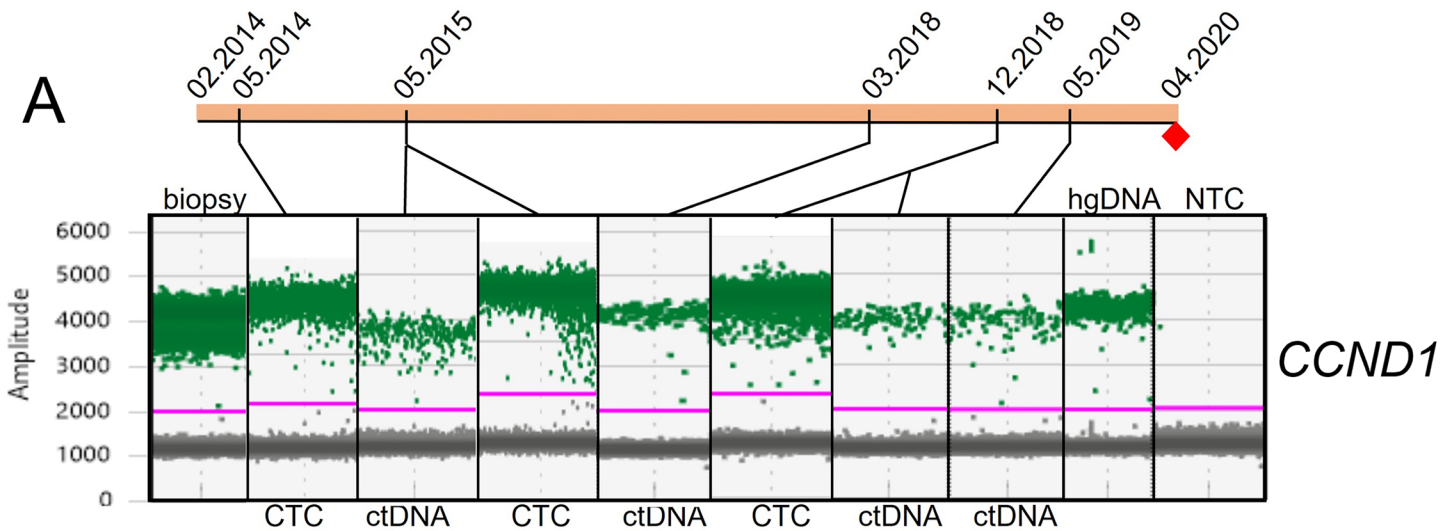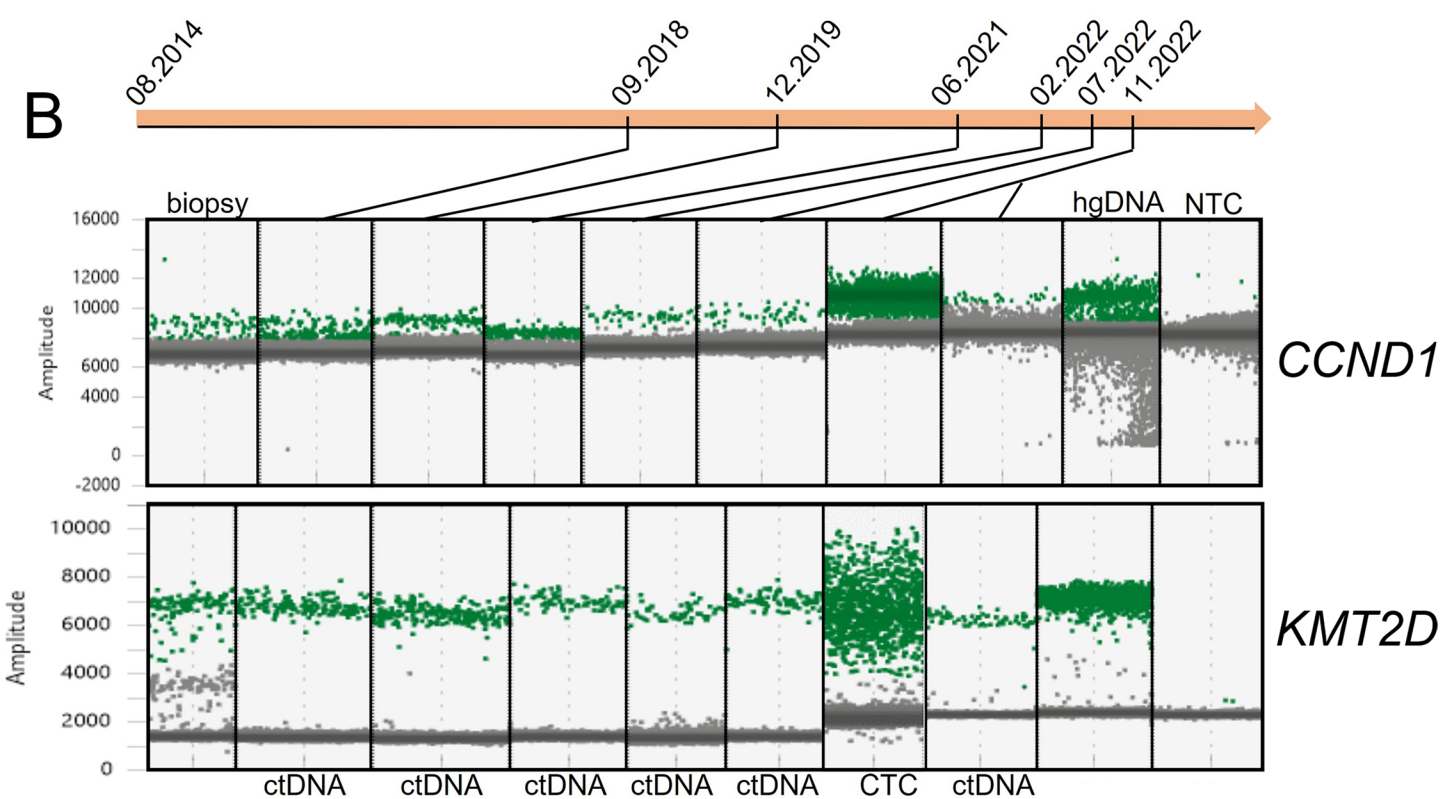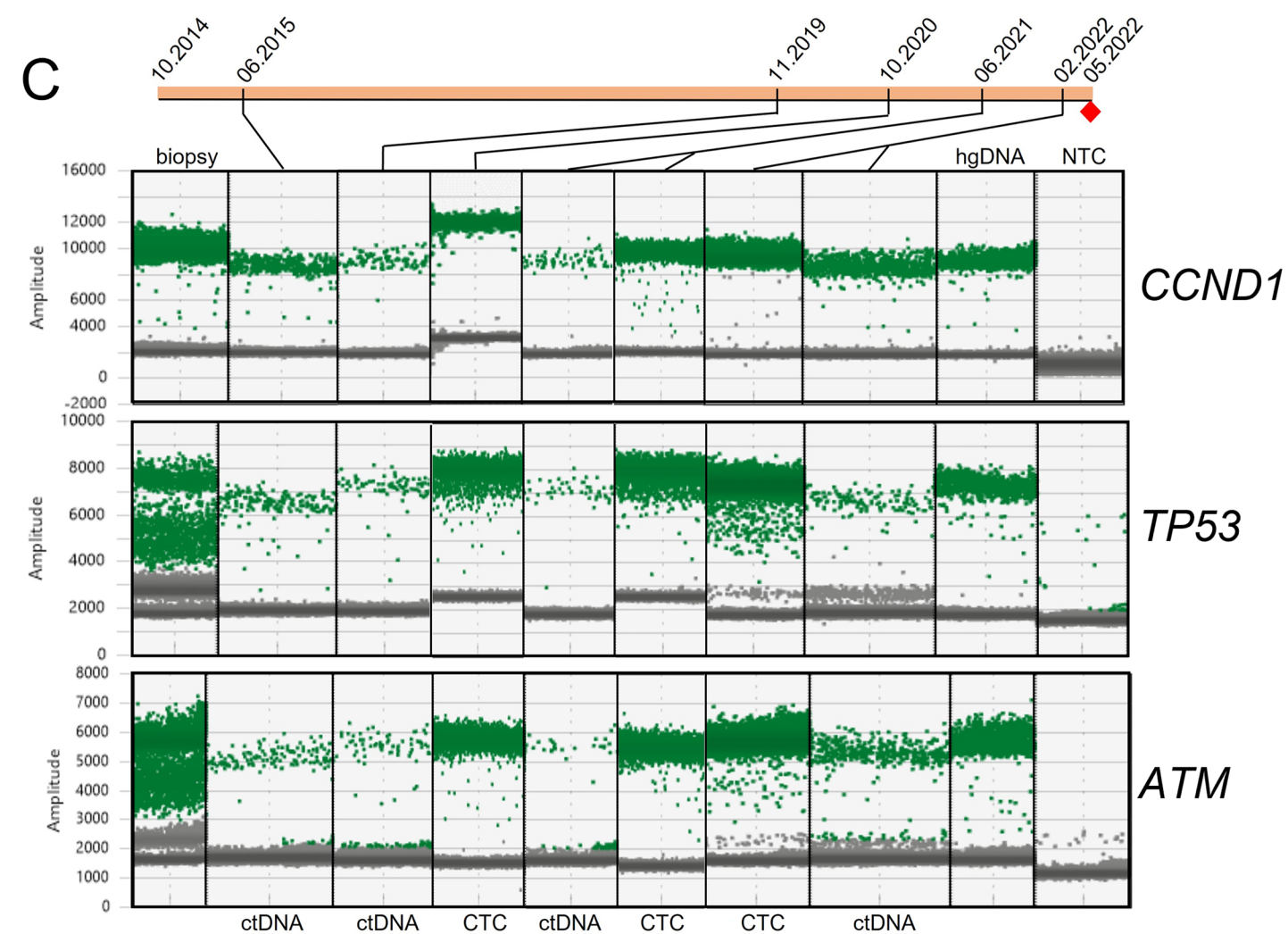

Supplement: Supplementary file 2 — FIGURE S2 Serial wild‐type ddPCR analysis was performed on CCND1 wildtype at the site of t(11;14) translocation breakpoint for patient 201‐139 (A), for patient 201‐162 (top panel) (B) and for patient 201‐170 (top panel) (C). Additionally, ddPCR analysis was performed for patient 201‐162 on KMT2D (bottom panel) (B), and for patient 201‐170 on TP53 (middle panel) and ATM (bottom panel) (C) corresponding wild‐type sequences with additional biopsy and human genomic DNA positive controls. Wildtype droplets are indicated in green; negative droplets are grey. NTC, no template control; ctDNA; circulating tumor DNA; CTC: circulating tumor cells. ddPCR assays were run at different time points and figures were constructed for clarity. [file JHA2-5-896-s001.pdf]
