# Supplementary material for: Monitoring of molecular responses to tirabrutinib in a cohort of exceptional responders with relapsed/refractory mantle cell lymphoma
Source: EJHaem. 2024 Jun 24;5(4):896–9. doi: 10.1002/jha2.966 (PMC11327755; doi:10.1002/jha2.966)
Supplement: Supplementary file 3 — Supporting Information [file JHA2-5-896-s004.docx]

**Supplementary materials and methods**

**Patient materials**

Patient samples were obtained after written informed consent. This study was approved by local Research Ethics Committee and the University Hospitals of Leicester NHS Trust (06/Q2501/122) and was conducted in accordance with Good Clinical Practice Guidelines and the Declaration of Helsinki. FFPE blocks were obtained from histology archive at Leicester Royal Infirmary. Peripheral blood mononuclear cells (PBMC) were separated using Ficoll histopaque (Sigma-Aldrich, St Louis, MO). Plasma was separated from blood following two consecutive high-speed centrifugations. gDNA from FFPE blocks was extracted using MagMAX FFPE DNA/RNA Ultra kit (Thermofisher, Waltham MA). gDNA from PBMC was extracted using the DNeasy Blood and Tissue kit (Qiagen, Hilden Germany). Cell-free DNA (cfDNA) was isolated from blood plasma using the QIAamp Circulating Nucleic Acid Kit (Qiagen, Hilden Germany).

Exome libraries were prepared using the SureSelect Human All Exon V6 kit (Agilent, Santa Clara, CA, USA), and paired-end sequenced on Illumina HiSeq4000 (2 x 150bp) to an average sequencing depth of 100X (Novogene, Cambridge, UK). FASTQ files were aligned with Burrow-Wheeler Aligner (BWA mem v0.7.17) to the human reference (NCBI build 37/hg19) genome build. Generated BAM files were sorted and indexed using Samtools v1.9. Base Quality Score Recalibration was performed using Genome Analysis Toolkit (GATK v4.0.3.0). PCR duplicates were identified using Picard v2.6.0. GATK Mutect2 v4.0.3.0 was used to call somatic mutations. Somatic copy number variations (CNVs) were called using GATK v4.0.3.0. Somatic mutations and CNVs were identified by paired analysis of tumor compared to matched germline controls. Moreover, variants present in GnomAD (v2.1.1) and ExAC with a frequency of more than 0.1% were filtered out to minimize artifacts related to germline contamination and sequencing error hotspots. Variants were also manually assessed in the Interactive Genomics Viewer (IGV) to confirm variant calls. Variants were annotated with VEP v99 and converted to MAF files for further analysis. Variant allele frequencies (VAFs) were calculated as the % of alternative reads (compared to the reference base call), normalized to the local copy number. In addition, WES data was used with Vidjil (1) to assess V(D)J recombination and mutation status. Raw FASTQ files were imported to the Vidjil web platform, and results interpreted in IGMT/HighV-QUEST. Chromosomal translocations were detected using FACTERA algorithm on aligned BAM files from resequencing with default parameters (2).

**ddPCR analysis**

ddPCR analysis of mutations and translocation breakpoints was performed using a Bio-Rad QX200 droplet digital PCR (ddPCR) system. Primers (Merck, Darmstadt, Germany) and probes (Applied Biosystems, Inchinnan, UK) were designed using Primer3 (Supplementary Table 3). Assays were run at optimal annealing temperatures. For all assays, no template controls were run to determine lack of contamination along with positive controls. BTK assays were run as in previously published (3).

**Flow cytometry**

The following monoclonal antibodies and reagents were used and were obtained from Beckman Coulter unless otherwise stated - Immunoglobulin λ light chain FITC (Dako, F0435), immunoglobulin κ light chain PE (Dako, R0436), CD20 PE-Cy7 (IM3629), CD19 Alexa-700 (B49212), CD38 BV421 (Becton Dickinson, 562444), CD45 Krome Orange (A96416), Tetrachrome-2 (6607073), CD8 Alexa-700 (B49181), CD4 PECy7 (737660), CD16 PE (A07766), Flowcount beads (7547053).  The data were acquired with a Navios flow cytometer and analysed with Kaluza software (v2.2.1). Serum immunoglobulins were quantified using a BNII (Siemens Healthineers) or an Optilite (The Binding Site) according to manufacturer’s instructions.

1. Duez M, Giraud M, Herbert R, Rocher T, Salson M, Thonier F. Vidjil: A Web Platform for Analysis of High-Throughput Repertoire Sequencing. PLoS One. 2016;11(11):e0166126.
2. Newman AM, Bratman SV, Stehr H, Lee LJ, Liu CL, Diehn M, et al. FACTERA: a practical method for the discovery of genomic rearrangements at breakpoint resolution. Bioinformatics. 2014;30(23):3390-3.
3. Jackson RA, Britton RG, Jayne S, Lehmann S, Cowley CM, Trethewey CS, et al. BTK mutations in patients with chronic lymphocytic leukemia receiving tirabrutinib. Blood Adv. 2023;7(14):3378-81.
