# Supplementary material for: Monitoring of molecular responses to tirabrutinib in a cohort of exceptional responders with relapsed/refractory mantle cell lymphoma
Source: EJHaem. 2024 Jun 24;5(4):896–9. doi: 10.1002/jha2.966 (PMC11327755; doi:10.1002/jha2.966)
Supplement: Supplementary file 4 — Supporting Information [file JHA2-5-896-s003.docx]

**Supplementary Tables**

Supplementary Table 1: Published outcome data in MCL with covalent BTKi.

| Covalent BTKi | Number of patients | Median PFS in months (95% CI) | Median OS in months (95% CI) | ORR (%) | CR (%) | PR (%) | Reference |
| --- | --- | --- | --- | --- | --- | --- | --- |
| Ibrutinib (Pooled analysis PCYC-1104, RAY, and SPARK) | 370 | 12.5 (9.8-16.6) | 26.7 (22.5–38.4) | 69.7 | 27.6 | 42.2 | (7) |
| Acalabrutinib | 124 | 20.0 (16.5-27.7) | Median not reached | 81 | 43 | 38 | (10) |
| Tirabrutinib | 16 | Estimated PFS 25.8 | Median not reached | 68.8 | 37.5 | 31.3 | (11) |
| Zanubrutinib | 86 | 33.3 (33.1-34.3) | Median not reached | 83.7 | 77.9 | 5.8 | (9) |

Supplementary Table 2. Mutational landscape prior to Tirabrutinib therapy detected by WES. Variant allele frequency (%) is shown in brackets.

| Group | Genes | MCL cases | | | |
| --- | --- | --- | --- | --- | --- |
|  |  | 201-139 | 201-162 | 201-170 |  |
| *IGHV* mutational status | *IGHV* | Unmutated (99.65%) | Unmutated (100%) | Unmutated (99.30%) |  |
|  |  | *IGHV4-34* | *IGHV3-23* | *IGHV4-34* |  |
| DNA damage response | *ATM* |  |  | p.L2427R (38%); p.Q2800P (38%) |  |
|  | *TP53* | p.R181H (5.6%) |  | p.L194R (85%) |  |
|  | *FBXW7* | p.R505C (4.6%) |  |  |  |
| Epigenetic and chromatin remodeling | *KMT2D* |  | p.S2773Lfs*72 (71%) |  |  |
|  | *SMARCA4* | p.P133A (49.5%) |  |  |  |
|  | *EZH1* |  | p.Y642F (40%) |  |  |
| NOTCH | *NOTCH1* |  |  | p.D2442Efs*35 (17%) |  |
|  | *NOTCH2* |  | p.P2303S (8%) |  |  |
|  | *NOTCH3* |  | p.L44M (9%) |  |  |
| Other | *RYR2* | p.C1489R (49%) |  |  |  |
|  | *UBR5* |  |  | p.E2702Kfs*16 (51%) |  |

Supplementary Table 3. Genes/variants and t(11;14) targets chosen for serial monitoring by ddPCR.

| **Patient Identifier** | **Target** | **t(11;14) breakpoint** | **Primers and probes sequences** |
| --- | --- | --- | --- |
| 201-139 | - | *CCND1;IGHJ4* | Forward mutated AGGAGAGAGGTTGTGAGGACT  Reverse mutated GCGTGGGATGAGATTAAACTGC  Forward CTAGTGGGAGGCAGGTGAAC  Reverse ACTGCGTCTTCTTCGTGGTT  Wild type CCTAGAGGGCTTTGTCCTACCATCCAG C  Mutated GGTGACCAGGGTTCCCTGGCCC |
| 201-162 | *KMT2D* (c.8315_8316insT) |  | Forward CCTCACCGGCTGTTCACAT  Reverse CAAGCTGAGTGGCCCCATC  Wild type CCGGGAGA**_**GTCGGTCATCGCTAG  Mutated CCGGGAGA**A**GTCGGTCATCGCTAG |
|  |  | *CCND1;IGHD* | Forward mutated GCAGGAGAGAGGTTGTGAGG  Reverse mutated AAACCACTTCCGACCACAGG  Forward CTGTGGTCGGAAGTGGTTTT  Reverse GGCTGGTACCCTGAGCAATA  Wild type CGGAAGGCCAGGGAATCTCT  Mutated AGAGGGCCGAACGTGACCTC |
| 201-170 | *TP53* (c.581T>G) |  | Forward CAGTTGCAAACCAGACCTCA  Reverse CAGGCCTCTGATTCCTCACT  Wild type TTCCACTCGGATA**A**GATGCTGAG  Mutated TTCCACTCGGATA**C**GATGCTGAG |
|  | *ATM* (c.8399A>C) |  | Forward TCAGCGAAGTGGTGTTCTTG  Reverse TGGGTGTCACTCACCATCAT  WT probe TTCAGTGCCTTTC**A**GTGCCAA  Mut probe TTCAGTGCCTTTC**C**GTGCC |
|  |  | *CCND1;IGHD2-15* | Forward mutated GAGGAAGCTCTAGGCAAAGG  Reverse mutated CAGCAGGAGAACAGACCAAA  Forward TTATCGGCTTGGATGTACTGAGG  Reverse AGCTTCCTCCAATTCACAAAGTC  Wild type AGAGGACCCACCCGTGGGGAGCT  Mutated TGGCTAATGTCATGTTACATACCTGCT |
